# Thidiazuron-induced somatic embryogenesis and changes of antioxidant properties in tissue cultures of half-high blueberry plants

**DOI:** 10.1038/s41598-018-35233-6

**Published:** 2018-11-19

**Authors:** Amrita Ghosh, Abir U. Igamberdiev, Samir C. Debnath

**Affiliations:** 1 Department of Biology, Memorial University of Newfoundland, St. John’s, Newfoundland and Labrador Canada; 2 St. John’s Research and Development Centre, Agriculture and Agri-Food Canada, St. John’s, Newfoundland and Labrador Canada

## Abstract

An efficient protocol of somatic embryogenesis (SE) has been developed for the first time in four half-high blueberry (*Vaccinium corymbosum* L. × *V. angustifolium* Ait.) cultivars. Thidiazuron (TDZ), a plant growth regulator with potential activities for shoot regeneration and shoot proliferation, was found most effective for somatic embryo formation when added to a nutrient medium at high concentration (9 µM). Although TDZ was also best for embryo germination at low concentration (2.3 µM), it was followed by zeatin at 4.6 µM for the same. Plantlets developed from SE were removed from the nutrient medium and transferred on a peat: perlite medium where 100% survival rate was acquired following the acclimatization process in a greenhouse. The concentrations of total phenolic and flavonoid contents were higher in greenhouse-grown conventionally cutting-propagated donor mother plants than those of respective SE plants for ‘St. Cloud’, ‘Patriot’ and ‘Northblue’ but not for ‘Chippewa’. The effect of propagation method and/or the older age of donor mother plants were clearly visible exclusively as the 15-year-old donor plants showed higher level of 2,2-diphenyl-1-picrylhydrazyl scavenging activity than the eight-weeks-old SE plants in all four cultivars.

## Introduction

Blueberry is a health-promoting small fruit crop, belonging to the genus *Vaccinium* L. (family: *Ericaceae*)^1^. It has very high commercial value and is cultivated across the world although the majority of the production is in the United States and Canada^2^. There are five different types of blueberries: northern highbush (*V. corymbosum* L.), southern highbush (species complex between *V. corymbosum and V. darrowii Camp*), lowbush (*V. angustifolium* Ait.), half-high (*V. Corymbosum* x *V. angustifolium*) and rabbiteye blueberries (*V. ashei Reade*)^1^. Half-high blueberries are hybrids between highbush (*V. corymbosum* L.) and lowbush blueberries^2^. These berries are greatly appreciated due to the superior quality and gained immense popularity among the consumers. Among 400–500 species in *Vaccinium* genus^1^, blueberries contain higher levels of phenolic compounds, which are high in antioxidant activities^3,4^. Presence of phenolic components in berry crops and their activities have been studied thoroughly due to the apparent relationship between the phytochemicals present in the plant product, and its association with the prevention of chronic diseases such as cancer, heart, vascular and neurodegenerative diseases^5–7^. Antioxidants act to prevent these diseases by scavenging free radicals^8,9^.

Although blueberry plants can be propagated conventionally by stem cutting, it is a labour-intensive and time consuming process. Micropropagation is a rapid and efficient method for mass propagation of blueberries which can be done all the year round. This can be obtained either by axillary bud proliferation and differentiation to mature plants or re-differentiation of newly formed meristematic tissues to fully grown plants^10^. There are two morphogenic pathways in plant regeneration: (1) organogenesis – the formation of root or shoot (2) somatic embryogenesis (SE) – formation of root and shoot meristem simultaneously^11^. In SE, somatic cells undergo a number of morphological and biochemical changes to form somatic embryos^12^. These include the formation of globular, oblong, heart, enlarge, torpedo, and cotyledonal-shaped structures^12^. The steps are: initiation, proliferation, maturation, and plantlets formation^13^. The SE is a well-recognized powerful tool for clonal propagation and has been explored in several agronomically and horticulturally important crop species^12^. The process can also be used for genetic transformation and artificial seed production^13^. Typically, the success of SE depends on explant types and the culture media containing an optimum plant growth regulator (PGR) regime. Based on the mode of occurrence, SE can be direct, without the intervention of callus stage or indirect SE, in which they are developed via callus phase^13^.

During past decades, thidiazuron (N-phenyl-N′-1,2,3-thiadiazol-5-ylurea) (TDZ) gained a lot of attention due to its prominent role on *in vitro* culture, with both auxin and cytokinin like effects, in different plant species^10,14^. Although limited reports on SE are available in few small fruit crops such as grape^15,16^ and strawberry^17^, none in detail with *Vaccinium* species. The present study aimed at whether somatic embryos can be regenerated *in vitro*, and the plantlet formation is possible from four half-high blueberry cultivars, in a TDZ containing medium. TDZ was also found effective to induce SE in grapes^16^ and for shoot proliferation and/or adventitious shoot regeneration of highbush^18^, lowbush^19^, and in half-high blueberries^18^.

*In vitro*-derived plants frequently show a phenotypic variation known as somaclonal variation^20^. This variation in tissue culture plants from their respective donor plants during the micropropagation is of significant important for commercial propagation and germplasm conservation^21^. The study also verified if there is any difference for the antioxidant properties between the SE plants and their corresponding donor mother plants. Investigation of the effects of embryogenesis on antioxidant properties in tissue culture plants compared to their respective donor plants has not been reported before in small fruit crops. However, this is of significant importance to establish this regeneration pathway as a reliable option of commercial blueberry production.

## Materials and Methods

### Plant material

The four half-high blueberry cultivars ‘St. Cloud’, ‘Patriot’, ‘Northblue’, and ‘Chippewa’, used for this study, were grown and maintained in a greenhouse of St. John’s Research and Development Centre, Agriculture and Agri-Food Canada (AAFC), St. John’s, Newfoundland and Labrador, Canada in plastic pots (10.5 × 10.5 × 12.5 – cm^3^) containing 3 peat: 1 perlite (v/v) under the natural light source of photosynthetic photon flux density (PPFD) at a maximum light intensity of 90 µmol m^−2^ s^−1^, 20 ± 2 °C at 85% of relative humidity with an automated sprinkler system for more than 15 years^18^.

### Effect of TDZ concentration on somatic embryo induction

Young and actively growing leaves of 2–3 weeks age were collected from the greenhouse-grown plants, surface sterilized^19^ and cultured on a blueberry basal medium (BM) containing three-quarter micro salts and macro salts of Debnath and McRae^22^ supplemented with (per litre) 3.5 g Sigma A 1296 agar, 25 g sucrose, and 1.25 g Gelrite™ (Sigma Chemical Co., St. Louis, MO, USA) for embryo induction. Leaves were placed, abaxial surface touching the medium, on 100 × 25 -mm Fisherbrand™ Petri dishes with clear lids (Fisher Scientific, Fair Lawn, NJ, USA) containing 25 ml of BM with 0 (control), 2.3, 4.5, 6.9, or 9 µM TDZ. The Petri dishes were then sealed along the rims with two layers of paraflim™. The pH of the medium was adjusted to 5.0 prior to the autoclaving at 121 °C for 20 min. TDZ was added in the medium before it was autoclaved. Each Petri dish contained five explants and three Petri plates were used per treatment. The cultures were placed in dark for 2 weeks at 20 ± 2 °C and then transferred to the diffused light (PPFD at 30 µmol m^−2^ s^−1^, 16 h photoperiod) provided by cool-white fluorescent lamps under same culture conditions. Data for somatic embryo formation were taken after 10 weeks of culture. The experiment was conducted three times within two-week-intervals. A 4 × 5 completely randomized factorial experiment was conducted to compare all treatment combinations of four half-high blueberry cultivars and 5 TDZ concentrations.

### Effect of type and concentrations of plant growth regulators (PGRs) on the maturation of somatic embryos

For embryo maturation and elongation of plantlets, 10-week-old somatic embryos developed on Perti plates with 9 µM TDZ, were transferred to 175-mL Sigma glass baby-food jars (Sigma Chemical Co., St. Louis, MO, USA) containing 35 mL BM supplemented with gibberellic acid (GA_3_) (0,1.4, 2.9, 4.2, or 5.8 µM), indole butyric acid (IBA) (0, 2.5, 4.9, 7.5, or 9.8 µM), Zeatin (ZEA) (0, 2.3, 4.6, 6.9, or 9 µM), or TDZ (0, 2.3, 4.5, 6.9, or 9 µM). Filter sterilized GA_3_ and ZEA were added in the cooled medium after autoclaving, while IBA and TDZ were added in the medium before autoclaving. Cultures were maintained under 16-h photoperiod at 30 µmol m^−2^ s^−1^ for shoot and root elongation. There were three jars per treatment and each jar contained five explants. The experiment was repeated three times. Data on embryo maturation and plantlet elongation was recorded after 10 weeks of transfer in glass jars. A completely randomized factorial experiment was laid out to compare all treatment combinations of four blueberry cultivars and, 16 PGR concentrations with controls (void of PGR).

### Acclimatization

After 8 weeks of culture, five plantlets (4–5 cm long) with 8–10 leaves developed on all concentrations of TDZ and ZEA, were removed from the glass jars, rinsed off in sterilized water, and planted in plastic pots (25 × 18 × 6; East-Chem Inc. Mount Pearl, NL, Canada) containing peat: perlite (3: 1 v/v). Plantlets developed on IBA or GA_3_ were not selected for acclimatization due to their poor vigour. The pots were maintained in a humidity chamber with a vaporizer at 20 ± 2 °C, 16-h photoperiod at 55 µmol/m^2^/s, humidity 95%. Acclimatization was performed by lowering down the humidity over 2–3 weeks to 85% which was found suitable for tissue culture-derived plantlet survival in a growth chamber^18^. Due to the improper development of cuticle, epicuticle and cuticular waxes and retarded functioning of stomatal apparatus during tissue culture process, *in vitro* grown plantlets at the time of acclimatization transpire in a high rate through the stomata and cuticles present on the leaves^23^. To avoid this excess water loss via the cuticle and stomata, micropropagated plantlets should be transferred slowly from high humid to low humid environment^23^. Data on survival rate of the plantlets were recorded after the hardening off process was complete (6 weeks). Hardened off plants were transferred in 6-cm^3^ plastic pots containing the same peat-perlite medium and grown in a greenhouse under natural light condition (temperature approx. 20 ± 2 °C, 16-h photoperiod, maximum PPFD = 90 µmol m^−2^ s^−1^, humidity approx. 85%)^18^.

### Extraction of polyphenolics from leaves of tissue culture and donor plants

The purpose of the biochemical assays was to compare the total phenolics, flavonoids, and antioxidant capacity in the leaves of SE plants with those of donor plants to investigate the effect of somatic embryogenesis and/or physiological age of plants on biochemical compounds, as well as to explore the potential of SE plants to synthesize phenolic and flavonoid compounds and evaluate the changes in antioxidant activity due to the use of TDZ in the growth medium during this process. Leaves collected from eight-weeks-old greenhouse-grown SE plants and more than 15-year-old four donor cultivars^24^ were shock-frozen, preserved immediately in liquid nitrogen and stored at −80 °C for chemical analyses. Antioxidant capacities in blueberries were found to be much higher in leaves than fruits^25^. At least three plants were selected randomly for each cultivar, and the leaves from each cultivar were collected in a replication of three from each plant. Five hundred milligrams of pre-frozen leaves from each plant were homogenized in a FastPrep-24 Tissue and Cell Homogenizer (MP Biomedicals, Irvine, CA, USA) in 80% aqueous acetone solution with 0.2% formic acid (1: 4 g/mL)^26^. The homogenate was kept for slow agitation at 4 °C for 30 min and then centrifuged at 13,000 rpm for 15 min at 4 °C using Allegra 64R (Beckman Coulter Inc., Palo Alto, CA, USA) before collecting the supernatant. Extraction was done two more times with the pallets and the supernatant was combined with the initial crude extract. The final volume of the crude extraction solution for polyphenolics was 6 mL. The extracts were preserved at ultralow freezer (Thermo Scientific, Burlington, ON, Canada) for further use to determine the contents of total phenolics, and flavonoids and the antioxidant capacity. All chemical analyses were carried out thrice with each sample and mean values were used for analysis.

### Determination of the total phenolic content

Total phenolic content was determined following photometric technique using the Folin-Ciocalteu method^27^, following Goyali *et al*.^26^. To obtain the final concentration 200 µg/mL, the crude extracts of polyphenolics were dissolved in methanol. Folin-Ciocalteu reagent (100 µL) was mixed to the diluted leaf extract (100 µL) and 20% saturated (w/v) sodium carbonate (200 µL) was added to it after 5 min. The mixture was added to 1.5 mL distilled water, kept in dark for 35 min at room temperature, and subjected to centrifugation at 4000 × g for 10 min in Allegra 64R. To identify the best wavelength, calibrated standards were processed according to the Folin-Ciocalteu index at 4 different wavelengths: 725 nm, 750 nm, 760 nm, and 765 nm using Ultrospec 4300 pro, UV/Visible Spectrophotometer (Amersham Biosciences Corp. San Francisco, CA, USA). Absorbance of the test samples were measured with UV/Visible Spectrophotometer at the wavelength of 725 nm, as according to mathematical calculations^28^, wavelength of 725 nm was found to be the best to determine Folin-Ciocalteu index^29,30^. Gallic acid solution with a concentration of 5 mg/mL with ≥98% purity was used as calibration solution. Linearity of the standard calibration curve for gallic acid was obtained in the range of 2.5–10 µg/mL and results were expressed as milligrams gallic acid equivalents (GAE) per gram of leaf weight (mg GAE/g lw).

### Determination of the total flavonoid content

Total flavonoid content of blueberry samples was determined according to colorimetric method^31^ following Goyali *et al*.^26^. 500 µL sample extract was added to the distilled water (2 mL) and 5% (w/v) sodium nitrate (150 µL). After 5 min, 150 µL of 10% (w/v) aluminium chloride was added to the mixture, incubated for 6 min at room temperature and 1 mL of 1 M sodium hydroxide solution was added to the mixture. To dilute the mixture, 1.2 mL distilled water was added to it and absorbance was measured at 510 nm using Ultrospec 4300 pro. Catechin solution (1 mg/mL) with ≥98% purity was used for standard curve calibration. As the flavonoid compound is present at a very high concentration and, anthocyanidins and flavonols are the most abundant flavonoids in blueberries^32^, and as reports are available that the main flavanol in blueberries is catechin^33^, it has been used as an ideal standard for this assay. Catechin has also been used by other researchers for the estimation of total flavonoid content in blueberries^34–36^. Linearity of the catechin standard calibration curve was obtained in the range of 20–200 µg/mL and results were expressed as milligrams catechin equivalents (CE) per gram leaf weight (mg CE/g lw).

### Determination of antioxidant activity

2,2-diphenyl-1-picrylhydrazyl (DPPH), an artificial stabilized free radical, was used to determine the antioxidant capacity^26^. The free radical scavenging activity was measured as percentage inhibition of DPPH radicals. 100 mL diluted extract was thoroughly mixed with 0.06 mM DPPH methanolic solution (1.7 mL) and incubated in dark for 60 min at room temperature and the absorbance of the mixture was monitored at 517 nm at 5 min interval to perform a saturation curve. Steady state of the DPPH reaction was observed at 45 min which was used to continue the experiment. Aqueous acetone (80%) mixed with DPPH solution was the blank. To calibrate the standard curve, gallic acid at 5 mg/mL (≥98% purity) was used. The linearity of the gallic acid standard curve was obtained in the range of 20–80 µg/mL and the results were expressed as mg GAE/g lw. Percentage inhibition was calculated using the following formula^37^:

$$\begin{array}{c} \% \,{\rm{Radical}}\,{\rm{scavenging}}\,{\rm{activity}}=[({{\rm{Absorbance}}}_{({\rm{Blank}})}-{{\rm{Absorbance}}}_{({\rm{Extract}})})\\ \,/{{\rm{Absorbance}}}_{({\rm{Blank}})}]\times 100\end{array}$$

### Statistical data analysis

All experiments were conducted following completely randomized design with three replications and the data were subjected to a two-way analysis of variance (ANOVA), employing general linear model for main effect ANOVA using STATISTICA version 10, data analysis software (Statsoft Wipro, East Brunswick, NJ, USA). Medium with no PGR did not show any response in terms of somatic embryo formation and were excluded from the analysis. Similarly, IBA at 2.5 µM was found ineffective for the maturation of somatic embryos in all four cultivars and the relevant data were also excluded from analysis. Tukey’s Test was used to compare treatment means at a critical difference (P) of ≤0.05. Results are presented as mean ± standard error. Correlation coefficient (r), coefficient of determination (r^2^) and linear regression between TPC and TFC, TPC and DPPH, TFC and DPPH were analysed using STATISTICA version 10 and the relationships were significant at a confidence interval 95% (α = 0.05).

## Results

### Effects of TDZ concentration on somatic embryo induction

**Figure 1:**
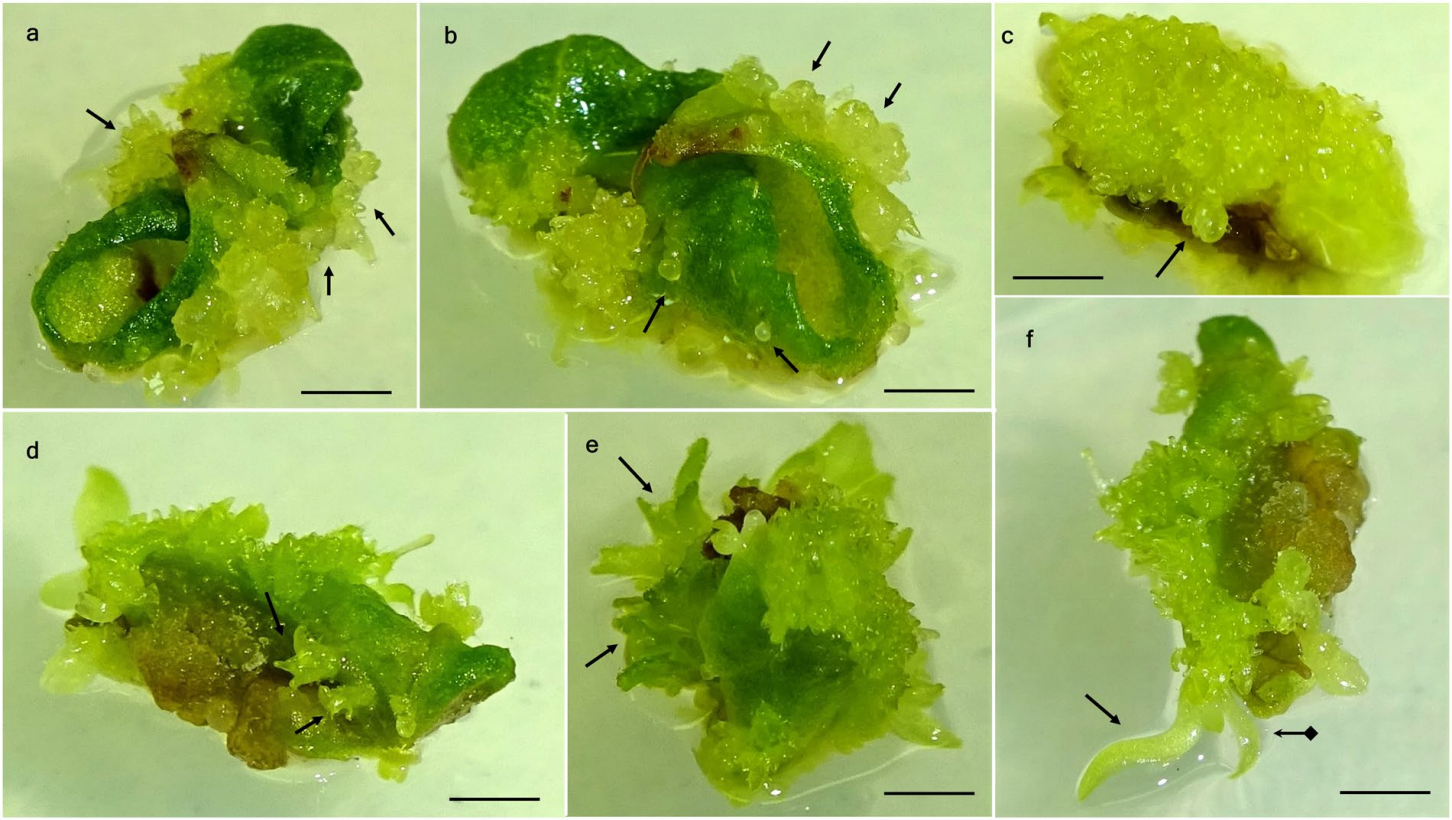
Somatic embryogenesis from leaf explants of ‘St. Cloud’ on a basal medium supplemented with 9.0 µM TDZ. **(a)** Formation of protuberances (arrows) after 2 weeks of incubation, **(b)** globular embryo development (arrows) from the protuberances after 4 weeks of culture, **(c)** heart-shaped embryo induction (arrow) after 6 weeks of culture, **(d)** torpedo-shaped somatic embryos (arrows) at 6 weeks in culture, **(e)** epicotyl development from embryos (arrows) after 8 weeks and **(f)** germination of somatic embryos and shoot (arrow with square end) and root apex development (arrow) after 10 weeks of culture. (Bars = 0.5 cm).

At all TDZ concentrations, within 2 weeks of incubation in dark, clear oblong-shaped protuberances started emerging at the edge of the leaves (Fig. 1a). Medium without TDZ did not show any response for embryo induction. Protuberances turned into globular shaped embryos within another 3–4 weeks of culture (Fig. 1b) under the diffused light. An additional week was needed for the formation of heart (Fig. 1c) or torpedo shaped embryos (Fig. 1d). Embryo formation was more at the edges of the leaf, than the middle part (Fig. 1b). Epicotyl development was observed within 7–8 weeks of culture initiation (Fig. 1e). Shoot and roots were formed from the meristem after another 2–3 weeks of culture (Fig. 1f).

**Figure 2:**
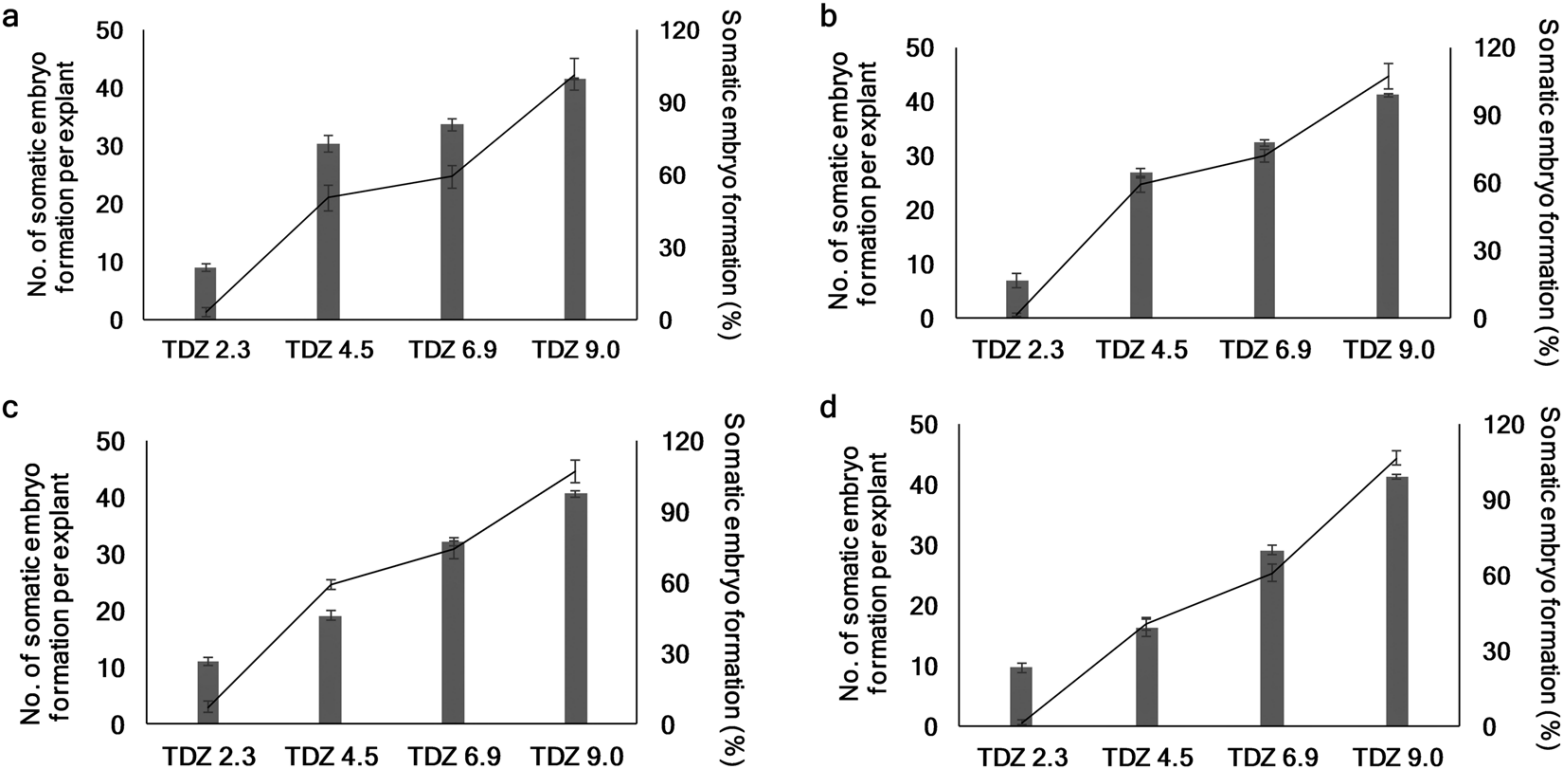
Effects of TDZ concentrations (µM) on the number of somatic embryos per explant (primary axis, line graph) and the percentage of embryo formation (secondary axis, column graph) in half-high blueberry cultivars after 10 weeks of culture. (**a**) ‘St. Cloud’, (**b**) ‘Patriot’, (**c**) ‘Northblue’, and (**d**) ‘Chippewa’. Each experiment was repeated three times. Tukey’s test was used to detect significant difference at P ≤ 0.05.

An interaction (P ≤ 0.05) between cultivar and TDZ concentrations was observed for the percentage of SE formation. The cultivars differed significantly (P ≤ 0.05) for this trait. Across TDZ concentrations, ‘St. Cloud’ (69%) was the best followed by ‘Patriot (64%), ‘Northblue’ (62%), and ‘Chippewa’ (58%). Across cultivars, highest percentage of embryo formation was observed at 9 µM (99%) and it was followed by 6.9 µM (76%), 4.5 µM (56%), and 2.3 µM (22%); and they were significantly different at P ≤ 0.05 from each other. At 2.3 µM of TDZ, the percent embryo formation values were the lowest in all four cultivars ranging from 17% to 27% for ‘Patriot and ‘Northblue’, respectively. The percentage of SE formation increased with the increasing concentrations of TDZ and at 9 µM TDZ, these values ranged from 98% to 100% in all four cultivars (Fig. 2).

ANOVA showed that only TDZ concentrations affected the number of somatic embryo formation (P ≤ 0.05). As was for the percentage of embryo formation, the number of embryos per explant also increased with the increase of TDZ concentration in all four cultivars (Fig. 2). While across cultivars, the frequency of embryo formation varied from 1.4 at 2.3 µM TDZ to 44 at 9 µM TDZ, the cultivars had 22–26 embryos per explant across all TDZ concentrations. At 9 µM TDZ, the number of embryos per explant varied from 42–45 in four cultivars.

### Effects of type and concentration of plant growth regulators (PGRs) on the maturation of somatic embryos

**Figure 3:**
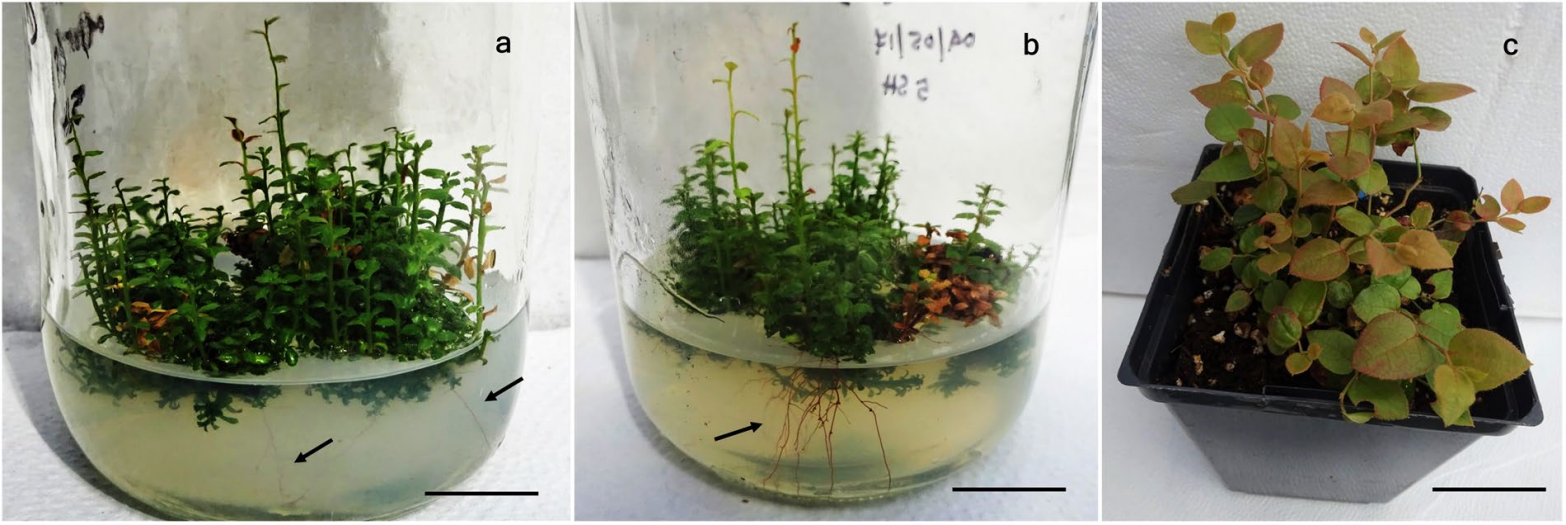
Shoot and root elongation and plantlet acclimatization of ‘St. Cloud’. **(a)** Root elongation (arrows) in a nutrient medium containing 2.3 µM of TDZ, after 4 weeks of culture in a glass jar. Bar = (2 cm). **(b)** Development of rooting system (arrows) in a nutrient medium with 2.3 µM of TDZ after 6 weeks of culture in a glass jar. (Bar = 2 cm). **(c)** Two-month-old greenhouse-grown plant in a 6-cm^3^ plastic pot containing peat-perlite medium. (Bar = 3.5 cm).

Somatic embryos with elongated shoots and apical root meristem developed on a BM with 9 µM TDZ, were transferred on BM supplemented with 5 different concentrations of GA_3_, IBA, ZEA, or TDZ to promote maturation. The medium without PGR and at 2.5 µM IBA was found ineffective for the maturation of somatic embryos in all four cultivars. The relevant results for rest of the treatment combinations are presented in Table 1. Analysis of variance revealed that the PGR type and concentration combinations affected significantly (P ≤ 0.05) the maturation of somatic embryos. GA_3_ at 1.4 µM promoted maturation of embryos only in ‘Chippewa’. All other PGR combinations enhanced embryo maturation in all four cultivars with the highest percentage being at 2.3 µM TDZ (27–31%) followed by 4.6 µM ZEA (20–23%). Maturation percentage of the embryos was very poor in BM with GA_3_ and IBA, it varied from 0–7% for GA_3_ and from 0–13% for IBA. Plantlets formed after maturation in BM containing GA_3_ and IBA were very poor in growth and eventually did not survive after more than 8 weeks of culture. Elongation of roots started after 4–5 weeks of culture (Fig. 3a) at 2.3 µM TDZ and 4.6 µM ZEA, and profuse rooting system was developed after 6–7 weeks of culture (Fig. 3b). After 8–9 weeks of culture in maturation medium, plantlets of 4–5 cm height were produced that were ready for transfer onto peat-perlite medium for acclimatization.

**Table 1:** Effect of different concentrations of PGRs on somatic embryo maturation and root elongation of four half-high blueberry cultivars.

PGRs concentration (µM)	Percent of somatic embryo maturation			
	‘St. Cloud’	‘Patriot’	‘Northblue’	‘Chippewa’
**GA**_**3**_				
1.4	0.00 ± 0.00 i	0.00 ± 0.00 h	0.00 ± 0.00 h	1.50 ± 0.29 g
2.9	1.11 ± 0.11 hi	1.78 ± 0.78 gh	1.83 ± 0.44 h	1.67 ± 0.67 g
4.2	4.33 ± 0.67 efghi	5.33 ± 1.45 fgh	3.44 ± 0.30 fgh	5.67 ± 1.20 fg
5.8	5.11 ± 1.06 efghi	4.17 ± 0.83 fgh	7.00 ± 1.15 defgh	7.00 ± 0.58 efg
**IBA**				
4.9	2.17 ± 0.60 ghi	3.00 ± 1.15 gh	2.44 ± 0.73 gh	1.83 ± 0.83 g
7.5	7.67 ± 1.20 defgh	10.00 ± 1.73 def	9.67 ± 1.45 cdefg	8.00 ± 1.15 defg
9.8	11.00 ± 1.53 cde	13.33 ± 0.88 cd	13.00 ± 1.00 cde	11.67 ± 0.88 cdef
**ZEA**				
2.3	15.00 ± 1.73 bc	16.89 ± 0.95 bc	10.56 ± 2.25 cdef	16.00 ± 1.00 bc
4.6	19.67 ± 1.45 b	22.67 ± 0.88 ab	20.67 ± 1.20 b	21.33 ± 0.67 b
6.9	8.67 ± 0.88 cdefg	7.00 ± 1.15 efg	9.33 ± 2.40 cdefg	7.33 ± 1.45 efg
9.2	4.00 ± 1.15 fghi	6.00 ± 0.58 fg	5.67 ± 0.88 efgh	3.00 ± 0.58 g
**TDZ**				
2.3	27.33 ± 1.20 a	27.67 ± 1.86 a	31.11 ± 1.25 a	29.33 ± 0.88 a
4.5	15.3 ± 2.03 bc	14.33 ± 0.67 cd	12.67 ± 2.40 cde	14.67 ± 2.60 bcd
6.9	13.00 ± 2.31 bcd	12.33 ± 1.45 cde	16.33 ± 1.76 bc	13.00 ± 2.31 cde
9.0	10.33 ± 0.88 cdef	9.67 ± 1.20 def	13.67 ± 1.45 bcd	12.00 ± 1.73 cdef

Each experiment was repeated three times. Maturation data was collected after 18 weeks of culture. Standard error associated with different letters indicates significant differences according to by Tukey’s test at P ≤ 0.05.

### Acclimatization

The plantlets when transferred to plastic pots containing a peat: perlite medium (3:1 v/v) and grown in a greenhouse where they acclimatized readily and all plants survived. Hardening-off plantlets have normal growth without any morphological variation (Fig. 3c).

### Total phenolic content (TPC)

The genotypes, propagation methods and their interactions affected significantly (P ≤ 0.05) the phenolic contents in cutting propagated donor and tissue culture plants (Table 2). Phenolic contents in blueberry plants followed the decreasing order of donor ‘Northblue’ (0.45 ± 0.00 mg GAE/g lw) > SE ‘Chippewa’ (0.43 ± 0.01 mg GAE/g lw) > donor ‘Chippewa’ (0.37 ± 0.01 mg GAE/g lw) and donor ‘Patriot’ (0.37 ± 0.02 mg GAE/g lw) > donor ‘St. Cloud’ and SE ‘Patriot’ (0.35 ± 0.01 mg GAE/g lw) > SE ‘St. Cloud’ (0.31 ± 0.00 mg GAE/g lw) > SE ‘Northblue’ (0.26 ± 0.00 mg GAE/g lw). ‘Chippewa’ tissue culture plants showed higher content of phenolics than those of the donor plants although in ‘St. Cloud’ and ‘Northblue’, donor plants had more phenolic contents. In ‘Patriot’, the greenhouse-grown donor plants and the SE plants had almost similar amount of total phenolics (Table 2).

**Table 2:** Effects of cultivar, and propagation method on total phenolic (TPC), flavonoid (TFC), and antioxidant activity (DPPH scavenging activity) of four half-high blueberry cultivars.

Parameters				
Cultivars (C)	Propagation method (P)	TPC (GAE/g lw)	TFC (CE/g lw)	DPPH scavenging activity (GAE/g lw)
‘St. Cloud’	D	0.35 ± 0.01 bc	9.97 ± 0.42 b	14.85 ± 0.70 a
	SE	0.31 ± 0.00 c	10.90 ± 0.10 ab	1.96 ± 0.23 c
‘Patriot’	D	0.37 ± 0.01 b	10.89 ± 0.06 ab	14.46 ± 0.32 a
	SE	0.35 ± 0.01 bc	8.03 ± 0.34 c	1.34 ± 0.10 cd
‘Northblue’	D	0.45 ± 0.00 a	10.89 ± 0.06 ab	15.05 ± 0.11 a
	SE	0.26 ± 0.00 d	4.88 ± 0.18 d	0.08 ± 0.00 d
‘Chippewa’	D	0.37 ± 0.02 b	7.93 ± 0.02 c	14.85 ± 0.18 a
	SE	0.43 ± 0.01 a	11.65 ± 0.70 a	6.48 ± 0.18 b
Significant effects		C, P, C × P	C, P, C × P	C, P, C × P

GAE = gallic acid equivalent, lw = leaf weight, CE = catechin equivalent, DPPH = 2,2-diphenyl-1-picrylhydrazyl radical, D = donor, SE = somatic embryogenesis. Data is expressed as means ± SE. Values with the different letters in the same column are significantly different at P ≤ 0.05 by Tukey’s test.

### Total flavonoid content (TFC)

From the results, it is evident that, not only the propagation method and genotypes, but also their interactions played a major role on the varying content of flavonoids present in the leaf extracts of the four half-high blueberry cultivars (Table 2). SE derived ‘Chippewa’ plants exhibited the highest TFC (11.65 ± 0.1 mg CE/g lw) followed by SE ‘St. Cloud’, donor ‘Northblue’, donor ‘St. Cloud’, SE ‘Patriot’, and donor ‘Chippewa’ while ‘Northblue’ tissue culture plants had the lowest TFC (4.88 ± 0.18 mg CE/g lw). Tissue culture plants had lower TFC than the donor plants in all cultivars except ‘St. Cloud’ where tissue culture plants had higher TFC than the donor plants (Table 2).

### Total antioxidant activity

Similarly, as TPC and TFC results, the genotype, propagation methods and their two-way interaction effects were significant (P ≤ 0.05) for DPPH (Table 2). In case of radical scavenging activity, greenhouse grown donor plants in all cultivars exhibited higher antioxidant activity than those of their respective SE counterparts. In this study, donor plants showed much high level of antioxidant activity ranging from 15.05 ± 0.11 (‘Northblue’) to 14.46 ± 0.32 (‘Patriot’) mg GAE/g lw, while leaves from SE regenerated plants displayed comparatively lower level of activity that varied from 6.48 ± 0.18 (‘Chippewa’) to 0.08 ± 0.00 (‘Northblue’) mg GAE/g lw (Table 2).

### Relationship among antioxidant properties

**Figure 4:**
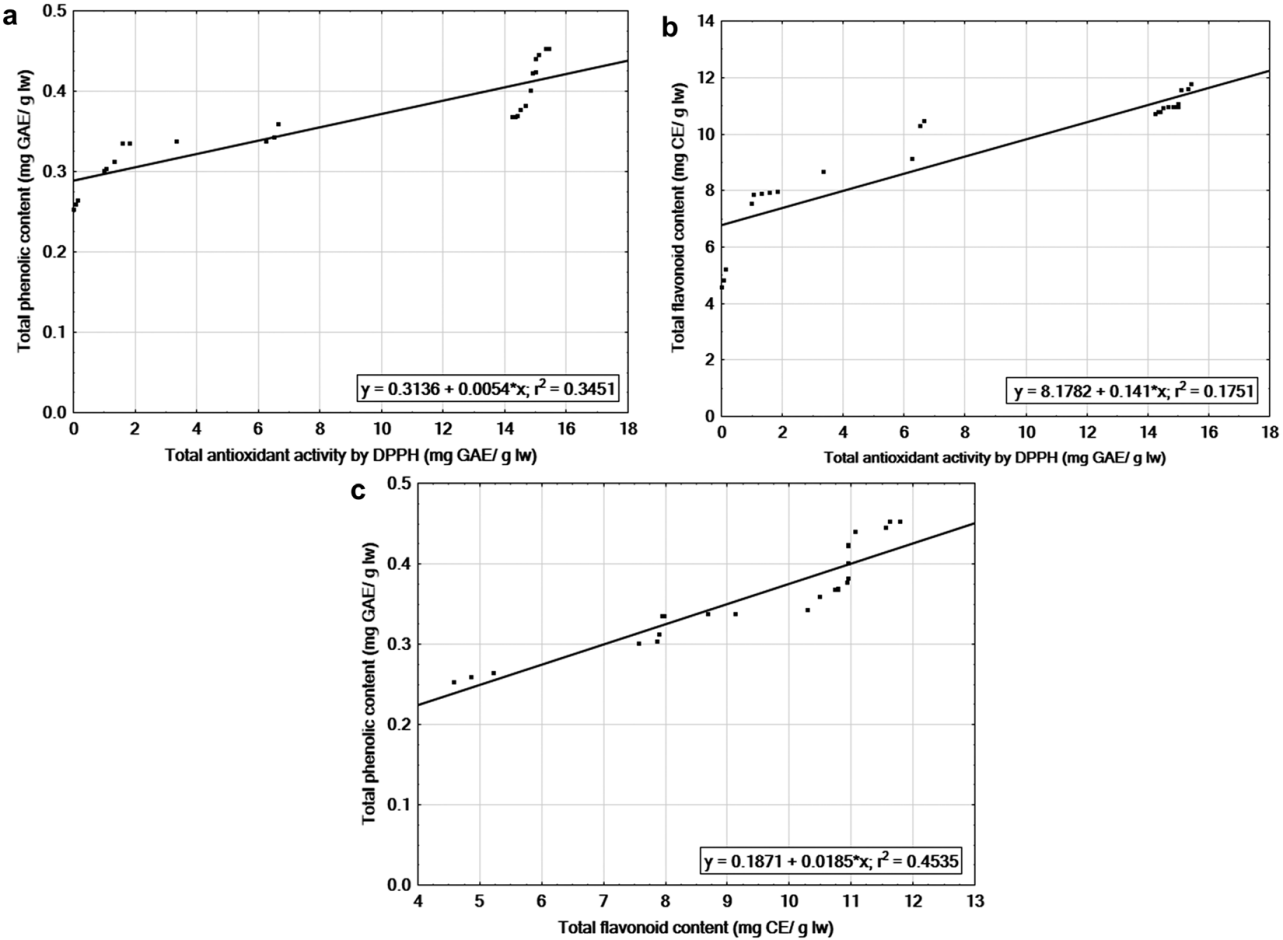
Linear regression between antioxidant properties in tissue culture and cutting propagated donor plants of four half-high blueberry cultivars. (**a**) Total antioxidant activity by DPPH (mg gallic acid equivalent (GAE)/g leaf weight (lw) and total phenolic content (mg GAE/g lw). (**b**) Antioxidant activity and total flavonoid content (mg catechin equivalent (CE)/g lw). (**c**) Total flavonoid (mg CE/g lw) and total phenolic contents (mg GAE/g lw).

The antioxidant activity determined by DPPH assay, had a significant linear correlation (r^2^ = 0.345) with TPC of the leaf extracts of four SE and donor plants. This shows that phenolic compounds of this extracts provide considerable amount of antioxidant activity. To evaluate the relationship between DPPH assay and TFC, linear regression was performed, which suggests a significant relationship between these two components (r^2^ = 0.175). The correlation coefficient was higher between antioxidant capacity and TPC (r) = 0.587, compared to antioxidant capacity and TFC (r = 0.418). TPC showed positive correlation with TFC (r^2^ = 0.453, r = 0.673) (Fig. 4).

## Discussion

This is the first report of SE in half-high blueberries. Some preliminary work on blueberry SE without any detail have been reported by Cui *et al*.^38^. In this study, TDZ was found effective for SE from leaf explants of half-high blueberries on a semi-solid medium. TDZ, although classified under the group of cytokinins due to its natural cytokinin-like response^39^, in *in vitro* culture, it exhibits both auxin and cytokinin-like activities in various plant species^14,40^. There are some hypotheses available on the mode of action of TDZ, but it is still unclear^41^. A possible explanation to the action mechanism of TDZ is that it may help in the accumulation and/or synthesis of endogenous plant growth hormones^40^. The promotion of growth such as callus formation, shoot regeneration, and somatic embryo formation take place when applied at higher concentration and axillary proliferation at comparatively lower concentrations which might be due to its similar biological activity like other N6-substituted cytokinins such as N-N-diphenylurea and N-(2-chloro-4-pyridyl)-N′-phenylurea^40,42^.

Among different TDZ concentrations, the highest number and percentage of somatic embryo formation was recorded in 9 µM TDZ after 10 weeks of culture. The same phenomena was also observed when TDZ was applied in higher concentrations (10–20 µM), giving rise to direct somatic embryos from the intact seedlings of pigeonpea instead of adventitious shoot regeneration^43^. Similarly, the use of TDZ in a range of 0.5–10 µM has been reported to induce SE in grape^16^ and in *Rubus*^44^. Somatic embryo development within 2 weeks of culture from the hypocotyl explants of geranium (*Pelargonium* × *hortorum*) was observed in the culture medium supplemented with 0.2–1 µM TDZ^45^. In *Saintpaulia ionantha* (H. Wendl.), TDZ in higher concentrations (5–10 µM) proved to be more effective for somatic embryo formation than the cytokinins benzyladenine (BA) and N-(2-chloro-4-pyridyl)-N′-phenylurea (CPPU) used in that study^46^. The percentage of embryo formation per explant was genotype dependent but in all four cultivars, increased SE was noticed with increasing the TDZ concentration. Similar results were observed during SE of 8 pigeonpea cultivars, where percent and average number of somatic embryo formation varied with genotypes, and embryo induction started at the higher concentration of TDZ (10–20 µM)^43^.

In the present study, maturation of somatic embryos was recorded on BM containing GA_3_ and IBA, but the plantlets were very poor in vigour and did not survive. Similar results were observed in geranium where exogenous application of GA_3_ on MS medium inhibited induction and expression of different SE stages and found to be detrimental on TDZ-derived SE formation^47^. IBA is a preferably used PGR for adventitious root induction from *in vitro* grown or cutting plants in woody plant species^48^. IBA was added in the medium prior to autoclaving as it is co-autoclavable with other media components, however it may lose some activity due to autoclaving^49^. IBA when added to the liquid medium before autoclaving approximately 20% loss of activity of IBA was observed in comparison to filter sterilization^49^. Additionally, IBA is sensitive to photooxidation and degrades in the tissue culture media during light incubation^49,50^. However, during incubation in dark for 28–30 days, IBA concentration decreases in agar solidified medium from 10–38% respectively^49,50^. Instability of IBA in light has several other effects on tissue culture system, which may be crucial during designing a plant tissue culture experiment^50^. It is also reported that direct SE from leaf explants of *Oncidium* ‘Gower Ramsey’ was completely inhibited when cultured on the medium supplemented with IBA^51^.

Our results demonstrated the effects of TDZ and ZEA on *in vitro* maturation of somatic embryo maturation in half-high blueberries. It has been reported that lower concentrations of cytokinin may initiate root formation while higher concentrations inhibit the rooting process and accumulate for shoot proliferation^52^. Although there is no report available on involvement of TDZ and ZEA on maturation of somatic embryos during multi-step embryogenesis, effect of these two PGRs are available on *in vitro* or *ex vitro* rooting of various plant species. For example, *in vitro* rooting with ZEA was recorded in Bounty’ strawberry (*Fragaria ananassa* Duch.); where sepal-derived adventitious shoots when cultured in the medium supplemented with 1–2 µM of ZEA, provide maximum rooting^53^. It was also reported that *in vitro* and *ex vitro* rooting can be obtained from the pre-treatment of microshoots of cranberry^54^, strawberry^55^ and blueberry^56^ in a TDZ containing medium. In soybean, the maximum number of root formation was reported from cotyledonary nodal explants in the B5 medium supplemented with 3.5–4.6 µM of TDZ^41^. Plantlets regenerated via SE were transferred to the greenhouse where complete survival was obtained after acclimatization. Similar results were also reported by Debnath^18^ where 80–90% survival rate was observed in *in vitro*-derived half-high blueberry plantlets when transferred to the greenhouse. In this study, the plantlets developed via SE without any intervention of callus phase, reduces the probability of occurrence of somaclonal variance to the minimum^43^.

Antioxidant properties of blueberries are well recognized due its medicinal importance to inhibit the detrimental effects on human health caused by free radicals. Plants can produce and accumulate health-promoting secondary metabolites in *in vitro* culture^57^. In this study, we have compared the changes of antioxidant properties among the cutting-propagated donor plants and SE regenerated plants. From the results of the biochemical assays, it was evident that propagation method and genotype have an impact on half-high blueberries under certain condition to synthesize phenols and flavonoids in the leaves. Leaf extracts from donor plants of ‘St. Cloud’ and ‘Northblue’ contains higher levels of TPC than SE plants, while donor plants of ‘Patriot’ did not show any significant difference than the SE counterparts. On the other hand, ‘Chippewa’ donor plants contain lower concentrations of TPC than the SE regenerated plants. The effects of the propagation methods on genotypes were also observed in the TFC profile, where SE regenerated ‘St. Cloud’ and ‘Chippewa’ plants showed higher level of TFC than greenhouse grown donor plants, and in ‘Patriot’ and ‘Northblue’ SE plants contains more TFC than mother plants. Plant tissue culture system has an important role on phenolic content biosynthesis, as the growth hormones used in the system may up or down-regulate the genes involved in the biosynthesis pathway^58^. Cytokinins found to be positively correlated with TPC, TFC, and condensed tannin concentration when applied individually or combined with auxin during micropropagation of *Aloe* species^59^. In lowbush blueberries, higher levels of polyphenols were reported in the leaves of conventionally grown plants than tissue culture (TC) plants, which could be due to the nutritional level difference affected by the propagation methods^29^. In that same study, it was also observed that the phytochemical profile of a wild lowbush clone was affected more by the propagation method than the cultivar ‘Fundy’^29^, which further supports the fact that propagation method and genotype have cumulative effects on the phytochemical components of the plant species. Phenolic compounds are the most abundant secondary plant metabolite derived from phenylalanine. Different environmental factors such as low light conditions and lower concentrations of nutrient increase the activity of phenylalanine ammonia lyase, which is a crucial regulatory factor of phenol metabolic pathway^60^. In the greenhouse, prolonged culture of plants could initiate low nutrient stress in the donor plant system which might act as an enhancer of TPC^29^.

We observed the changes of antioxidant properties in the TDZ-induced SE plants. Although there is no report on comparative study of antioxidant properties between SE and donor plants, drop in TPC level was noticed during SE in cacao^61^. In another study, no direct relationships between presence and absence of polyphenolics with embryogenic or non-embryogenic condition of cacao callus were detected but presence of TPC and tannins were observed during somatic embryogenic response^62^. In *Artemisia absinthium* callus cultures, production of phenolic contents was positively related with the presence of TDZ and cultures with lower concentration (0.5–3.0 mg/L) displayed maximum TPC and antioxidant potential^63^.

The determination of antioxidant activity is a complex procedure as it is a combination of synergistic and antagonistic effects of various environmental factors^64^.To measure the antioxidant activity of DPPH radical scavenging method was used, which is a widely used, rapid, and easy method to detect antioxidants^65^. DPPH is a stable free radicle, when dissolved in ethanol it produces a violet solution and in the presence of antioxidant molecules it is reduced to a uncoloured solution^65^. The propagation method as well as the physiological age had a clear effect on the antioxidant activity of the four cultivars, as all donor plants showed significantly higher levels of activity than SE plants. These results align with the TPC profile of ‘St. Cloud’ and ‘Northblue’, and TFC profile of ‘Patriot’ and ‘Northblue’ where the leaves of SE plants from the same cultivar contained more polyphenols and flavonoids. This indicates that TPC and TFC may not adequately explain the antioxidant activity of the leaf extracts, which are admixture of different components with diverse activities. Additionally, total antioxidant activity represented by the DPPH value is the sum of various antioxidant compounds, which also relies on the chemicals used during the extraction process from the leaves^30^. Similarly, in lingonberry (*V. vitis-idaea* L. ssp. *vitis-idaea* Britton) higher antioxidant activity in the leaves of greenhouse-grown cutting propagated plants in comparison to the TC plants were reported earlier, although in fruits of the same species, contained higher phenolic and anthocyanin components than the cutting plants^66^. The reasons behind the presence of higher content of antioxidant in the conventionally grown plants are not very clear, but the possible reason could be the age of the plants as well as the culture condition. In the present study, the donor plants were more than 15-years-old, while the SE plants were eight-weeks-old. Although reports on the effects of the age of tissue culture and donor plants on antioxidant properties are lacking, young leaves of various varieties of blackberries, raspberries and strawberries were found to contain higher TPC and antioxidant activity than the older leaves^67^. Similar results were observed in tea leaves, where comparatively younger leaves showed higher ORAC values and contained more polyphenols predominantly (–) -epigallocatechin 3-galate than the older leaves^68^.

We observed positive correlation between antioxidant activity with the total phenolic and flavonoid content of the SE and donor plants in four cultivars. Various studies on blueberries showed a significant positive correlation between TPC profile and antioxidant capacity^3,26,29^ and of TFC with antioxidant properties, evaluated by DPPH radical scavenging activity^26,29^. Our results showed that antioxidant activity measured by DPPH assay were slightly better correlated to total phenolics than to total flavonoids components. These results are in agreement with the previous findings on blueberries^3,29^.

In conclusion, this study, for the first time, reported SE in half-high blueberries and the changes in antioxidant properties due to this process in the tissue culture regenerated plants. Results of this study indicate that these findings could lead to tremendous labour and time savings and SE can be used as a useful means of commercial blueberry propagation.
